# The Microbiota and Allergies/Asthma

**DOI:** 10.1371/journal.ppat.1000549

**Published:** 2010-05-27

**Authors:** Gary B. Huffnagle

**Affiliations:** Pulmonary Division, Department of Internal Medicine, University of Michigan, Ann Arbor, Michigan, United States of America; University of California San Francisco, United States of America

## What Is the Evidence Linking Changes in the Microbiota to the Development of Allergic Disease?

Two lines of evidence suggest that environmental changes are a major factor in the development of allergies: the increase in the incidence of allergic diseases over the past 20–30 years and the dichotomy in the rate of allergic disease between industrialized and developing countries. These observations have led researchers to propose the “hygiene hypothesis” for allergies and asthma. Simply stated, a lack of early microbial stimulation results in aberrant immune responses to innocuous antigens later in life [1]. However, an alternative interpretation of the evidence supporting the hygiene hypothesis forms the foundation of the “microbiota (microflora) hypothesis” [2], [3]. The concept is that significant perturbations in gastrointestinal (GI) microbiota composition in westernized areas (due to antibiotic use, dietary changes, and other lifestyle differences) have disrupted the mechanisms of mucosal immunologic tolerance. Epidemiologic and clinical data supporting this interpretation include 1) a positive correlation between increasing risk for asthma/allergies and increasing use antibiotics in industrialized countries, 2) correlations between altered fecal microbiota composition and atopic disease, and 3) successful prevention/reduction of allergies in some individuals by oral probiotics or dietary changes. Experimental data in mice include the observations that 1) germ-free animals display numerous defects in immune response generation, 2) antibiotic treatment can augment Th2 type immune responses, and 3) probiotics can diminish airway allergic responses. Altogether, these experimental, epidemiologic, and clinical observations support the hypothesis that changes in the indigenous microbiota can be a predisposing factor for allergic disease.

## How Stable Is the Microbiota and Can It Be Altered?

The mucosal surfaces in the body are the home to the indigenous microbiota that, in humans, is estimated to be composed of 10–100 trillion microbes, with a diversity of greater than 1,000 species [4], [5]. The highest concentration of microbes is found in the GI tract, while extremely low numbers are found in the airways. Healthy human lungs are not sterile [6], as previously believed [7]–[8], but it is unknown whether the microbes in the lungs form a stable community or are a series of transient colonizers.

Throughout the rest of the mucosa, the microbiota at each site resides as a stable climax community, which is defined as a microbial community that has reached a final or “climax” steady state as a result of a series of ecological successions that have selected species best adapted for growth at that specific niche along the mucosa. However, this climax community is dynamic and still exhibits both resistance and resilience [9], [10], i.e., it has the ability to maintain a given community structure in the face of perturbation, but is also able to return to its baseline structure following environmental perturbation after resistance is broken. Evidence is now accumulating that long-term dietary pressures and repeated antibiotic use can break both the resistance and resilience of a community and result in it re-assembling into another climax community [11], [12], although this may be accompanied by detrimental changes in host mucosal immunobiology and physiology. Other environmental pressures on the indigenous microbiota can include GI illnesses or medications such as antacids, proton pump inhibitors, and non-steroidal anti-inflammatory drugs. One mechanism underlying the activity of probiotic microbes and prebiotic nutrients may be the ability to restructure a climax community to improve host mucosal immunobiology and physiology.

## How Are Microbiota Changes and Mucosal Immune Responses in the Gut Linked to Mucosal Immunity in the Lungs?

The mucociliary architecture of the nasopharyngeal cavity and upper airways naturally sweeps all inhaled micro-particulates that stick to the mucus lining into the GI tract. Shortly after intranasal inoculation, fluids, particles, and microbes introduced into the nasal cavity are largely found in the GI tract [2]. Thus, inhaled micro-particulates (which comprise the vast majority of aeroallergens) are also swallowed. For example, in one animal model of allergic airway disease, two days after intranasal administration of antigenic peptide, corresponding Ag-specific CD4 T cell division had not only occurred in the lymph nodes draining the lungs and nasopharyngeal cavity, but also in the mesenteric lymph nodes [13]. No division was seen in peripheral non-draining nodes.

The propensity of ingested antigens to block subsequent systemic immune responses is termed oral tolerance [14]. It is likely that oral tolerance and airway tolerance are tightly linked and the GI tract acts as a “sensor” for the development of tolerance to inhaled and injected antigens. The results of depletion, reconstitution, and adoptive transfer studies have demonstrated that tolerance to low amounts of ingested allergens is mediated by CD4+ regulatory T cells (Tregs). The mechanisms of Treg-mediated suppression are not entirely known, but it is clear that Tregs require T cell receptor stimulation and that production of immunosuppressive cytokines, IL-10 and TGFβ, are critical mediators in vivo [15]. Thus, Tregs require specific activation but can mediate nonspecific suppression in what is termed “bystander suppression.” Interactions between dendritic cells and T cells are also central to mucosal tolerance and mucosal signals, such as those from the microbiota, that keep resident dendritic cells in an immature or non-inflammatory state will promote the development of Tregs. This could be true for all mucosal sites. Our hypothesis is that this “sensor” system for mucosal tolerances can be modified by genetics (affecting innate immune cells) and to an even greater extent by perturbations of microbiota signaling exerted by antibiotics and significant dietary changes. While generation of Tregs is one mechanism, there are likely a number of others that may involve humoral immunity, innate mechanisms, and potentially even neurogenic pathways [16]. It remains to be determined how these distal mucosal sites interact in generating mucosal immunity and what the microbial signals are that promote tolerance, although they are likely to include short chain fatty acids and zwitterionic polysaccharides [17], [18].

## What Is the Role of Indigenous Yeast in This Cross-Kingdom Signaling in the Mucosa?

*Candida albicans* and other *Candida* species are a normal part of the human microbiota and reside in low numbers in the mouth, vagina, and GI tract of healthy individuals. The composition of the microbiota, hormones, stress, innate immunity, and adaptive immunity are all factors that impact the levels of *Candida* colonization. Increased levels of *Candida* species in the microbiota have been implicated for decades in a number of hypersensitivity diseases, although a definitive mechanistic understanding has been lacking [19]. *C. albicans* colonization of the GI mucosa has been implicated at some level in 1) atopic dermatitis, a chronic inflammatory skin disease; 2) celiac disease, an allergic/autoimmune reaction to gluten; 3) Crohn's disease, an inflammatory bowel disease in which anti–*Saccharomyces cerevisiae* antibodies (ASCA) develop that are reactive to a cell wall epitope of *C. albicans* that is expressed in vivo but not in standard culture; and 4) “fungal-type dysbiosis” (reported in the popular media as “yeast syndrome”), a controversial diagnosis defined as multiple manifestations of a diverse collection of syndromes, including food sensitivities, allergic responses, digestive problems, and psychoneurological manifestations.

We have demonstrated that colonization of mice by *C. albicans* following broad-spectrum antibiotic therapy (cefoperazone) can promote the development of allergic airway disease [20], [21]. These responses were maximal in mice that received both antibiotic treatment and oral introduction of *C. albicans*, implicating a need for a change in both the bacterial and fungal microbiota to promote the development of allergic disease. Other studies have demonstrated that extraluminal leak of antigen is greater in *C. albicans*–colonized mice than in *C. albicans*–free mice [22]. The mechanisms underlying these observations are still under investigation, but *Candida* species (and many other fungi) can secrete prostaglandins and prostaglandin-like molecules de novo or via conversion of exogenous arachidonic acid [23]. Prostaglandins are potent immunomodulatory molecules that can promote Th2 type responses and tissue eosinophilia. Fungal cell wall glucans are also powerful inflammatory stimulants in tissues and may also play a role in the immunomodulatory activity of yeast in the GI tract. Finally, there is antagonism between *Candida* and members of the indigenous microbiota, which may impact bacterial–host immunoregulatory responses in the mucosa.

## Can the Microbiota Be Targeted for Therapy of Allergic Disease and, If So, How?

The composition of the microbiota can be manipulated by combinations of antibiotics, probiotics, and dietary components. Probiotics are defined as live microbes that, when delivered in sufficient quantities, exert a beneficial effect on health [24], [25]. Probiotic consumption has been practiced for over a century and has resulted in a large body of anecdotal evidence that suggests a connection to improved health. Fortunately, these are being replaced by clinical studies and mechanistic investigations that are demonstrating positive results for probiotics, both therapeutically and preventatively. Many dietary components also have direct growth promoting or inhibiting activity for specific microbes, such as certain types of fatty acids, phenolic compounds, and carbohydrates. However, a single type of probiotic or dietary component will not be efficacious in all individuals. This likely owes to differences in the types of microbial communities in different individuals. The objective of the international Human Microbiome Project is to characterize and define the human microbiome in states of health and disease [26]. The challenge for future research is to use this information to optimize probiotic/dietary therapy to improve human health and prevent microbiota-associated diseases, such as allergies.
